# Prevalence and Pattern of Refractive Errors among Primary School Children in Al Hassa, Saudi Arabia

**DOI:** 10.5539/gjhs.v5n1p125

**Published:** 2012-11-11

**Authors:** Fahd Abdullah Al Wadaani, Tarek Tawfik Amin, Ayub Ali, Ataur Rahman Khan

**Affiliations:** 1 Department of Surgery, Ophthalmology section, College of Medicine, King Faisal University, Saudi Arabia; 2 Community Medicine and Public Health, Faculty of Medicine, Cairo University, Cairo, Egypt; 3 Public Health Practice & Administration, Institute of Public Health Lahore, Pakistan; 4 Department of Family & Community Medicine, College of Medicine King Faisal University, Saudi Arabia; 5 Primary Health Care, Local Health Directorate, Al Hassa, Saudi Arabia

**Keywords:** refractive errors, primary schoolchildren, prevalence, Saudi Arabia

## Abstract

Some 12.8 million in the age group 5–15 years are visually impaired from uncorrected or inadequately corrected refractive errors. In Saudi Arabia, the size of this public health problem is not well defined especially among primary schoolchildren. The purpose of this cross-sectional study was to assess the prevalence and pattern of refractive errors among primary school children in Al Hassa, Saudi Arabia. A total of 2246 Saudi primary school children aged 6 to 14 years of both genders were selected using multistage sampling method form 30 primary schools located in the three different areas of Al Hassa. School children were interviewed to collect demographics and vision data using a special data collection form followed by screening for refractive errors by trained optometrists within the school premises using a standardized protocol. Assessment of visual acuity and ocular motility evaluation were carried out and cover-uncover test was performed. Children detected with defective vision were referred for further examination employing subjective refraction with auto refractometer and objective refraction using streak retinoscopy after 1% cyclopentolate. Of the screened school children (N=2002), the overall prevalence of refractive errors was 13.7% (n=274), higher among females (Odds ratio, OR=1.39, P=0.012) and significantly more among students of rural residence (OR=2.40, P=0.001). The prevalence of refractive errors was disproportionately more among those aged 12-14 years (OR=9.02, P=0.001). Only 9.4% of students with poor vision were wore spectacles for correction. Myopia was the most commonly encountered refractive error among both genders (65.7% of the total errors encountered). Uncorrected refractive errors affected a sizable portion of primary school children in Al Hassa, Saudi Arabia. Primary schoolchildren especially females, rural and older children represents high risk group for refractive errors for which the included children were unaware.

## 1. Introduction

Visual impairment due to refractive errors is one of the most common childhood problems and the second leading cause of treatable blindness (Dandona & Dandona, 2001). It is estimated that globally 153 million people over 5 years of age are visually impaired as a result of uncorrected refractive errors, of whom 8 million are blind (WHO, 2006). Furthermore, some 12.8 million in the age group 5–15 years are visually impaired from uncorrected or inadequately corrected refractive errors, a global prevalence of 0.96%, with the highest prevalence reported in urban and highly developed urban areas in south-east Asia and in China (Zhao et al., 2004). Vision disorders are the fourth most common disability of children and the leading cause of handicapping conditions in childhood (Murry & Lopez, 1996; Ciner et al., 1998). Visual impairment from uncorrected refractive errors can have immediate and long-term consequences in children and adults, such as lost educational and employment opportunities, lost economic gain for individuals, families and societies, and impaired quality of life (Resnikoff et al., 2004; Larry et al., 1997). Various factors are responsible for refractive errors remaining uncorrected: lack of awareness and recognition of the problem at personal and family level, as well as at community and public health level; non-availability of and/or inability to afford refractive services for testing; insufficient provision of affordable corrective lenses; and cultural disincentives to compliance (WHO, 1993). In the age group 5–15 years, non-correction of refractive errors is due to several factors: the lack of screening, and the availability and affordability of refractive corrections are the most important. However, cultural disincentives also play a role, as shown in surveys from countries where routine screening and provision of corrections are free of charge or easily accessible, but compliance remains low (Preslan & Novak, 1998; Khandekar et al., 2002). A remarkable finding of one study showed that even in economically advantaged societies, refractive errors can go undetected or uncorrected in children (Vitale et al., 2006). In Saudi Arabia, reliable studies that tackle the prevalence and pattern of refractive errors among primary school children are scarce, with varied results due to inconsistency in the cutoff used for errors definition form 23% in the high altitude Abha city (Abolfotouh et al., 1993) to 10.7% among pre-school children in Jeddah city at sea level (Wedad et al., 2002). Moreover, the Saudi school health services provided by ministry of education do not include adequate vision screening facilities (Wedad et al., 2002). In accordance with WHO’s global initiative “Vision 2020” The right to sight (http://www.vision2020.org/main.cfm?type=WHATVISION2020), a professional based (optometry) screening program for all school-aged children is recommended to provide an early detection and initiate early treatment. The objective of the this study was determine the prevalence and pattern of refractive errors among primary school children in the age group of 6-14 years of both sexes in Al Hassa, Eastern Province, Saudi Arabia.

## 2. Material and Methods

### 2.1 Setting and Design

This is a cross-sectional study that was carried out in Al Hassa Governorate, Eastern Province of Saudi Arabia; located 50 km from the Arabian Gulf, 450 km from the capital Riyadh, and populated by about 1.5 million. Al Hassa is comprised of three regions; urban (mainly included Al Hofuf and Mubrraz cities), populated by about 60% of the total population, rural (called Al Omran with aggregate of several villages) consisting of 23 villages (35% of the population) and “Hegar” Bedouin scattered communities making up the remaining 5%. The Ministry of Health provides primary care through 54 PHCs, while the rest of the population are provided with primary care through other sectors e.g., National Guard, ARAMCO (oil company), military and others. School health services are provided free of charges to students in Al Hassa, through two specialized centers one each in Hofuf and in Al Omran, both providing curative/preventive primary care with referral system to the higher levels.

### 2.2 Sampling and Sample Size

Epi-Info version 2002 (CDC, Atlanta, GA, U.S.A) was used to calculate the required sample size. For this cross-sectional study design the following considerations were employed for sample size calculation: the total recorded population for the academic year 2011 was 181045 (Al Hassa Directorate of School Health records, 2011). Assuming a prevalence of refractive errors of %11 (Wedad et al., 2002) and the worst acceptable prevalence of 9%, applying a margin of error of 5% (95% confidence), the sample size would be 936. A design effect of 2 was considered in employing the cluster method of sampling, hence the sample size accounted to 1872. Twenty percent contingency factor was added, taking into account non responders. Thus, the final sample size was 2246 school children. A proportionate sampling method was applied with regard to the rural–urban distribution using an appropriate sampling fraction. Sampling was carried out using a multistage sampling method, first we divided Al Hassa into the three main areas for sample selection namely Al Hofuf, Mubrraz and Al Omran and the representation was proportional to the number of children enrolled in primary schools in each area (estimated to be 690 children from Omran, 696 from Hofuf and 860 from Mobarraz). An updated list of all public primary schools was used as the sample frame, and a total of 30 primary schools (10 from each division consisting of 5 Girls’ and 5 boys’ schools) were selected for this study by simple random method. Finally, the third phase was the recruitment of children aged between 6-14 years from each participating school. The class registers were used and systematic random sampling was done. Primary school children aged 6-14 years old who did not have any history of eye injuries or eye disease (e.g. corneal opacity, cataract or retinal pathology) affecting visual function were legible for inclusion.

### 2.3 Methods

#### 2.3.1 Demographics and Vision Data

A special form was used to collect socio-demographic and personal including name, age, sex, history about present and past ocular problems and treatment and using of spectacles by students, the siblings using spectacles, results of visual acuity tests and the presence of squint.

#### 2.3.2 Examination for Refractive Errors

We followed the study protocol supported by WHO named ’The Refractive Error Study in Children (RESC)’ by using consistent definitions and methods (Négrel et al., 2000).

Legible school children were first screened at the school premises by the optometrists and nurses specially trained for Refractive Error Study. A standard ophthalmic examination procedure was used for each study subject. Ophthalmic examination included assessment of visual acuity for distance with Snellen’s illiterate ‘E’ chart at room illumination and ocular motility evaluation. The distant vision of a child was tested with the chart at 6 meters Hirschberg test was performed to find out the presence of squint. A cover-uncover test was performed to confirm the diagnosis if strabismus was detected.

Children detected with defective vision were referred for further examination at three permanent ophthalmology clinics situated at the three sectors PHCs; Al Omran, Al Hofuf and Al Mubarraz. Each sector PHC has a well-established ophthalmology clinic. Referred students were further evaluated employing subjective refraction with the help of auto refractometer. In some younger children objective refraction was performed with streak retinoscopy after 1% cyclopentolate drop instilled in each eye for at least half an hour prior examination.

#### 2.3.3 Pilot and Filed Testing

The study field staffs included eight nurses (4 females and 4 males) specially trained in vision screening, two optometrists, two ophthalmologists and one coordinator. Survey fieldwork was preceded by 2 weeks of staff training and all field staffs were familiarized with the standard examination procedures involved. A 5-day field exercise was performed (in a nearby two primary schools beyond those included in the final sampling) to validate the data collection and to minimize inter-observer variations. The inter-rater agreement for refractive errors (any) ranged from 93-100%. For the two ophthalmologists, the inter-rater agreement for the presence of refractive errors ranged from 95-100%.

#### 2.3.4 Definitions of Variables

**Presenting vision** is the visual acuity in the better eye with the currently available refractive correction, if any.

**Best corrected Vision** is the visual activity in the better eye achieved either by pin hole or by refraction.

**Visual impairment** is the visual acuity of less than 6/6 in the better eye but if it is < 6/18 in the better eye it must be improved to equal to or better than 6/18 by refraction or pinhole thus spanning the low vision and blindness categories as currently defined in the ICD-10.

**Myopia** is defined as a spherical equivalent refractive error of at least -0.75 D in one or both eyes.

**Hypermetropia** is defined as a spherical equivalent refractive error of at least +2.00 D or more in one or both eyes.

**Astigmatism** is defined (as cylinder powers ≥0.50 DC or ≥1.00 DC) if one or both eyes were astigmatic.

**Amblyopia** is defined when binocular optimal visual acuity is subnormal even after full refractive correction. Crowding phenomenon and titmus fly teat were also used to detect amblyopia.

**Emmetropic eye** is defined if neither eye is myopic or hypermetropia.

## 3. Data Analysis

Data analysis was done using SPSS version 17.0 (SPSS Inc. Chicago, Ill, U.S.A). Prevalence of visual impairment (visual acuity 6/12 or worse) and blindness (visual acuity of < 6/60) was calculated for uncorrected visual acuity, baseline (presenting) visual acuity, and best measured visual acuity. The latter measurement was based on subjective refraction obtained in those with reduced uncorrected visual acuity. Association of refractive error with age, sex and geographical area was explored by using Chi square test, trend analysis and Fisher Exact test.

## 4. Ethical Consideration

The study protocol was approved by the ethics committee at King Faisal University. Permissions form the Al Hassa Education Directorate and the principals of selected schools were obtained. Written consent forms from parents/legal guardians were required as a prerequisite for inclusion. Assents from selected school children were also obtained. The results of child’s examination were sent to schools using a special reporting format for subsequent management by school health authorities.

## 5. Results

Of the 2246 primary school children approached, 2002 were included with approved consent forms from thier parents/guardians with a response rate of 89.1%. Those refused to participate were not significantly different in regard to their demographic characteristics.

### 5.1 Demographics

The age of the included school children ranged from 6 to 15 years with a mean of 9.4 years (SD=2.3) and a median of 9.5 years. Table 1 depicts the socio-demographic characteristics of the included primary school children. Urban school children represented 71.9%, females constituted 51.7% and 88.3% were in the age group <12 years while only 11.7% were in the age range of 12 to 14 years. Grades one and six were less compared to other school grades (13.8% for grade one and 12.7% for grade six). One percent of the included children worn spectacles, the figure were reported among their siblings.

**Table 1:** Demographic characteristics of the included primary school children, Al Hassa, Saudi Arabia (N=2002)

Characteristics	Number	%
**Gender**		
Male	966	48.3
Female	1036	51.7
**Residence**		
Rural	561	28.0
Urban	1441	72.0
**Age groups (years)**		
6-<9	870	43.5
9-<12	897	44.8
12-14	235	11.7
**Mean Age**	9.48±2.31	
**School grades**		
1	277	13.8
2	359	17.9
3	399	19.9
4	353	17.6
5	360	18.1
6	254	12.7
**Children using spectacles**	21	1.0
**Spectacles among siblings**	20	1.0

### 5.2 Prevalence of Refractive Errors

The overall prevalence of refractive error among the primary school children was 13.7% (n=274) (95% Confidence Intervals ’C.I’ =12.2%-15.2%), higher among females (n=161) than males (15.5% vs. 11.7%, Odds ratio, OR=1.39, P=0.012) and significantly more among students of rural residence (123/561, 23.71%) compared to urban (151/1441, 10.5%), (OR=2.40, P=0.001). Bilateral errors were encountered in 226 (82.6%) of the children detected with refractive error, followed by right eye (10.7%) and left eye (6.7%).

The prevalence of refractive errors was disproportionately more among those aged 12- 14 years (47.5%, CI= 45.3-49.6%, OR=9.02, P=0.001), compared to 9.8% and 8.6% in those aged 6-8 and 9-11 years respectively (Table 2). Only 9.4% (26/274) of students with poor vision were wore spectacles for correction. About 9.0% of the siblings of the children detected with refractive errors were wearing spectacles.

**Table 2:** Distribution of refractive error in relation different socio-demographic variables (N=2002)

Variables	Refractive errors: No. (%)		Odds ratio (95% C.I) and P value

	Present (N=274)	Absent (N=1728)	
**Gender:**		
Male	113 (11.7)	853(89.1)	Reference
Female	161(15.5)	875(88.5)	1.39(1.06-1.81)*
**Residence:**		
Rural	123(21.9)	438(78.1)	2.40(1.83-3.14)**
Urban	151(10.5)	1290(89.5)	Reference
**Age Groups:**			
6-<9 years	85(9.8)	785(90.2)	Reference
9-<12 years	77(8.6)	820(91.8)	0.43(0.32-.058)**
12-14 years	112(47.5)	123(52.3)	9.02(6.59-12.35)**
**School grade:**			
1	23(8.3)	254(91.7)	1.00†
2	37(10.3)	322(89.7)	1.27
3	33(8.3)	366(91.7)	1.00
4	31(8.8)	322(91.2)	1.06
5	79(21.9)	281(78.1)	3.10
6	71(28.0)	183(72.0)	4.28l**

C.I= Confidence intervals,

* P value <0.05,

** P value<0.001,

† Chi-square for trend.

### 5.3 Types of Refractive Errors

Table 3 displays the different types of refractive errors encountered among primary school children distributed in relation to gender, residence and age groups.

**Table 3:** Distribution of types of refractive errors by socio-demographic characteristics among the included primary school children

Refractive errors: No. (%)						

Variables	Myopia (Spherical≥-0.75D)(N=180)	Myopic Astigmatism (Cylindrical ≥0.75)(N=34)	Hypermetropia (Spherical ≥ +2 D)(N=27)	Hypermetropic astigmatism (Cylindrical≥0.75)(N=33)	Total (N=274)	P value*
**Gender:**						
Males	70(25.5)	16(5.8)	14(5.1)	13(4.7)	113(41.2)
Females	110(40.1)	18(6.6)	13(4.7)	20(7.3)	161(58.8)	0.014
P value **	0.010	0.973	0.854	0.394	161(58.8)
**Residence and Gender:**						
**Rural:**						
Male	27(9.9)	4(1.5)	4(1.5)	4(1.5)	39 (14.2)
Female	69(25.2)	9(3.3)	3(1.1)	3(1.1)	84(30.7)	0.001
**Urban**						
Male	43 (15.7)	12(4.4)	10(3.7)	9(3.3)	74(27.0)	0.001
Female	41 (15.0)	9(3.3)	10(3.7)	17(6.2)	77(28.1)
P value **	0.001	0.252	0.977	0.494	
**Age groups:**						
6-<9	63(23.0)	5(1.8)	13(4.7)	4(1.5	85(31.0)
9-<12	53(19.3)	17(6.2)	5(1.8)	2(0.7)	77(28.1)	0.001
12-14	64(23.4)	12(4.4)	9(3.3)	27(9.9)	112(40.9)
P value †	0.001	0.001	0.188	--

* Chi square for independence.

** Z test for proportions,

† Chi square for trend.

Myopia was the most commonly encountered refractive error among both genders (65.7%). The prevalence of myopia accounted to 9.0 % (CI =7.7-10.2%) hypermetropia was detected in 27 students (1.4%, CI= 0.80-1.9%), followed by myopic 34 (1.7%, CI =0.10-2.3%) and hyperopic astigmatism in 33 (1.7%, CI=1.1% to 2.2%) in the population studied.

The prevalence of myopia, myopic astigmatism, hypermetropia and hypermetropic astigmatism among those with refractive errors were accounted for 65.7%, 12.4%, 9.9% and 12.1% respectively. Simple myopia, myopic astigmatism and hypermetropic astigmatism were more prevalent among females (31.3%, 6.6% and 7.3% compared to 26.8%, 5.8% and 4.7% in males respectively) while males had more hypermetropia (5.1% vs. 4.7%) than the female. Female students with rural residence were more affected compared to urban females, the prevalence of refractive errors was accounted to 30.7% (CI=28.8-32.8%) compared to 28.1%, (CI=26.6-30.6%) among urban (P= 0.001). Also, females from rural areas were more affected with myopia as compared to urban females (25.2%, CI=23.3-27.1% vs. 15.0%, CI=13.4-16.5%). Hypermetropia and hypermetropic astigmatism were more prevalent among urban than rural female (3.7%, CI=2.8-4.5% vs. 1.1%, CI=0.6-1.5% for hypermetropia, and 6.2% CI=5.2%-7.3% compared to 1.1%, CI 0.6-1.5% for hypermetropic astigmatism).

Male students with urban residence had high frequency of myopia (15.7%, CI=14.1-17.3% compared to 9.9 %, CI=8.5-11.2% among rural males). Astigmatism was significantly more among females than males (13.9%, CI=12.4-15.4% vs. 10.6%, CI=9.2-11.9%). Urban females had high prevalence of astigmatism compared to rural females (9.5%, CI=8.2-10.8% compared to 4.4%, CI=3.5-5.3%). There was an incremental relationship observed between myopia and increasing age in studies population.

Myopia was more common (23.4%, CI=21.5-25.2%) in the older age group (12-14 years) than in younger age groups of 6-8 years and 9-11 years where its frequency was 23.0% and 19.3% respectively. Older age group also showed increased prevalence of both myopic astigmatism (4.4%) and hypermetropic astigmatism (9.9%) compared to younger age group. However hypermetropia was more common (4.7%) among the younger age group of 6-8 years as compared to the older age groups of 9-11 years (1.8%) and 12-14 years (3.3%).

Amblyopia was found in 29 (1.4%) of the study population. Amblyopic factors among the children with amblyopia were anisometropia 21 (1.0%) and strabismus 8 (0.4%).

## 6. Discussion

Visual impairment from uncorrected refractive errors can have immediate and long-term consequences in children and adults, such as lost educational and employment opportunities, lost economic gain for individuals, families and societies, and impaired quality of life (Resnikoff et al., 2004). Various factors are responsible for refractive errors remaining uncorrected: lack of awareness and recognition of the problem at personal and family level, as well as at community and public health level; non-availability of and/or inability to afford refractive services for testing; insufficient provision of affordable corrective lenses; and cultural disincentives to compliance (WHO, 2003).

The estimate of visual impairment caused by uncorrected refractive errors is of public health concern (Resikoff et al., 2004; Thylefors, 1998; Dandona & Dandona., 2001) despite that refractive errors could be easily diagnosed and that spectacle correction is among the most cost-effective interventions in eye care. According to Baltussen et al. (2008), screening of 5–15 years old yields the most health effects and more absolute terms, both screening of 10–15 years and 5–15 years old are very cost-effective strategies. Therefore screening of the school children is an important measure to know the magnitude of refractive error and their correction at the appropriate time.

In this study 13.7% of the screened primary school children were positive for uncorrected refractive errors. The prevalence of refractive errors among our sample of primary school children which was higher than that reported form similar study conducted in Saudi Arabia (AI Rowaily & Alanizi, 2010) of 9.8% among intermediate school students, Malaysia, 7.7% (Hashim et al., 2008), Nepal 8.6% (Pokharel et al., 2010), Iran 3.5% (Fotouhi et al., 2007), Uganda 11.6% (Kawuma & Mayeku, 2002) and Bangkok 12.7% (Yingyong, 2010) and lower than those reported from Qatar of 19.7% (AL-Nuaimi et al., 2008), and India (Padhye et al., 2009) of 25.1% [Table 4].

**Table 4:** Prevalence of refractive errors among school children in different parts of the world

Country	Year	Age group	Sample size	Prevalence of refractive errors
**Iran**	2007	6-17	5544	3.8%
**Malaysia**	2008	6-12	840	7.7%
**Nepal**	2011	5-16	2236	8.6%
**Uganda**	2002	6-9	623	11.6%
**Bangkok**	2010	6-12	1100	12.7%
**India**	2007	6-12	2317	25.1%
**Qatar**	2010	6-13	670	19.7%
**Saudi Arabia**	2010	12-13	1536	9.8%
**Saudi Arabia Current**	2011	6-14	2002	13.7%

This variation may be related to the type of sampling method used, size of population screened and the variation of geographical location in these studies. Unlike other studies where higher prevalence of refractive error has been documented in urban population (Pokharel et al., 2010; Fotouhi et al., 2007; Kawuma & Mayeku, 2002; AL-Nuaimi et al., 2010; Padhye et al., 2009), our study has found higher prevalence of refractive among rural schoolchildren. However Ahuama and Atowa (1987) in their population based study in Uganda had found a higher prevalence of refractive error in rural area than urban (29.0% compared to 21.6% among urban students).

Higher prevalence of refractive errors in rural area may be attributed to the rapid urbanization in Saudi Arabia (Basha, 1992) with greater access to and abundance of computers and electronic gadgets as a result of advanced socio-economic transition coupled with the availability and regularity of electricity which have motivated children to remain indoors and involved in activities which cause more eye strain.

We have found a higher prevalence of refractive error among female students than males. This is consistent with similar studies carried out in Riyadh, Saudi Arabia among intermediate school students where it was reported a prevalence of 11.7% among females compared to 8.3% among males (AI Rowaily & Alanizi, 2010), similar findings were reported form Qatar (AL-Nuaimi et al., 2008) with 23.7% prevalence of refractive errors among females compared to 15.5% among males), India (Prema, 2011) with 17.2% vs. 13.4% among males, Ghana (Ovenseri-Ogbomo & Assien, 2010) and Germany (Jobke et al., 2008).

Of the encountered refractive errors in this study, myopia was the leading type found representing 65.7% which is consistent with other studies done in various parts of the world. The research conducted in Malaysia (Hashim et al., 2008), Nepal (Pokharel et al., 2010), India (Padhye et al., 2009), Jordan (Bataineh & Khatatbeh, 2009) and Qatar (Al-Naimi et al., 2010) have found that myopia represented 77.5%, 59.8%, 20.65%, 31.05%, 63.5% and 25.54% of screened errors respectively among 6-14 years schoolchildren.

In our study the rate of myopia was significantly higher in rural children. This is in contrast to other studies where the prevalence of myopia has been found to be higher in urban area than rural (Hashim et al., 2008; Pokharel et al., 2010; Padhye et al., 2009). This may be due to different definitions of myopia used in these studies (<-.50D). We used ->0.75D as the myopic definition. Other reason for this increased myopia in rural area may be due to decreased outdoor activities in rural children than the urban. Nevertheless, for the age group 5–15 years, the prevalence of visual impairment from uncorrected refractive errors in some regions appears to be higher in urban areas than in rural areas, despite the reported better access to services. This may be due to a high incidence of myopia in these populations: it is suggested that there may be a direct cause–effect relation between increased access to education and myopia, but other secular changes could be contributing factors (Resnikoff et al., 2004).

During the last three decades Saudi Arabia has witnessed an increasing mushroom growth of parks, super malls and gyms in all urban areas including Al Hassa, thereby increasing the outdoor recreational activities for urban children. Studies form Australia, Singapore, United States and Turkey has shown that outdoor activities act as a protective factor for myopia (Rose et al., 2008; Ip et al., 2008; Onal et al., 2007; Mutti et al., 2002; Jones et al., 2007). It must be mentioned that the students were screened for distant vision only hence, number of hypermetropia is far less than myopic ones.

In the current study we have found a high prevalence of astigmatism (24.5%), similar results have been reported form Qatar (70%) (Al-Naimi et al., 2010), Ghana (49.3%) (Ovenseri-Ogbomo & Assien, 2010), Pakistan (35.5%) (Ali et al., 2007) and Jordan (20.4%) (Bataineh & Khatatbeh 2008) and contrary to those found in Nepal (9.2%) (Pokharel, 2010) and China (8.3%) (Rose et al., 2010).

These findings warrant the urgent implementation of fundamental policies including screening of children for refractive errors that should be conducted at community level and integrated into school health programs, accompanied by education and awareness campaigns to ensure that the corrections are used and cultural barriers to compliance are addressed and removed. Corrections must be accessible and affordable for people of all age especially those at school age. Training and information program should also be tailored for teachers and school health-care workers. There is a need to conduct this type of study in different parts of Saudi Arabia to know national magnitude of the refractive error which will help the health authority to formulate appropriate strategy for effective screening program throughout the country.

## 7. Study Limitations

The potential effects of the encountered errors in the form of scholastic achievements were not studied; also the possible risk factors responsible for the development of the different types of errors were not possible due to difficulties inherent with the used research design.

## 8. Conclusion

Uncorrected refractive errors affected a sizable portion of primary school children in Al Hassa, Saudi Arabia. Primary schoolchildren especially females and rural children represents high risk group for refractive errors for which the included children were unaware. Periodic screening in schools should be carried out; school teachers, children and their parents should be educated about signs and symptoms of refractive errors and for the risk factors involved in their development.
